# Mesenchymal stromal cell delivery of full-length tumor necrosis factor–related apoptosis-inducing ligand is superior to soluble type for cancer therapy

**DOI:** 10.1016/j.jcyt.2015.03.603

**Published:** 2015-07

**Authors:** ZhengQiang Yuan, Krishna K. Kolluri, Elizabeth K. Sage, Kate H.C. Gowers, Sam M. Janes

**Affiliations:** Lungs for Living Research Centre, UCL Respiratory, Division of Medicine, University College London, London, United Kingdom

**Keywords:** apoptosis, cancer, mesenchymal stromal cell, TRAIL, tumor

## Abstract

**Background aims:**

Mesenchymal stromal cell (MSC) delivery of pro-apoptotic tumor necrosis factor (TNF)-related apoptosis-inducing ligand (TRAIL) is an attractive strategy for anticancer therapy. MSCs expressing full-length human TRAIL (flT) or its soluble form (sT) have previously been shown to be effective for cancer killing. However, a comparison between the two forms has never been performed, leaving it unclear which approach is most effective. This study addresses the issue for the possible clinical application of TRAIL-expressing MSCs in the future.

**Methods:**

MSCs were transduced with lentiviruses expressing flT or an isoleucine zipper-fused sT. TRAIL expression was examined and cancer cell apoptosis was measured after treatment with transduced MSCs or with MSC-derived soluble TRAIL.

**Results:**

The transduction does not adversely affect cell phenotype. The sT-transduced MSCs (MSC-sT) secrete abundant levels of soluble TRAIL but do not present the protein on the cell surface. Interestingly, the flT-transduced MSCs (MSC-flT) not only express cell-surface TRAIL but also release flT into medium. These cells were examined for inducing apoptosis in 20 cancer cell lines. MSC-sT cells showed very limited effects. By contrast, MSC-flT cells demonstrated high cancer cell-killing efficiency. More importantly, MSC-flT cells can overcome some cancer cell resistance to recombinant TRAIL. In addition, both cell surface flT and secreted flT are functional for inducing apoptosis. The secreted flT was found to have higher cancer cell-killing capacity than either recombinant TRAIL or MSC-secreted sT.

**Conclusions:**

These observations demonstrate that MSC delivery of flT is superior to MSC delivery of sT for cancer therapy.

## Introduction

Cancers are one of the leading causes of human death in the world. Each year, more than 10 million new cases of cancer occur globally. Current treatments for metastatic cancers including chemotherapies or radiotherapies often provide limited benefits to patients and are frequently accompanied by undesired side effects. Novel therapies are needed.

Tumor necrosis factor (TNF)-related apoptosis-inducing ligand (TRAIL) is a promising agent for cancer therapy. TRAIL is a type II transmembrane protein with homology to other members in the TNF family [1,2], and it selectively triggers apoptosis in tumor cells while sparing normal cells [1–3]. It is safe to deliver, with the ligand exhibiting no detectable cytotoxicity to normal tissues in murine and primate models [4,5] or in humans [6]. Agonist monoclonal antibodies to TRAIL receptors (TRAIL-Rs) have also been used in phase II clinical trials and have shown good safety and tolerability [5,7,8]. However, the enthusiasm in developing TRAIL as a novel cancer therapeutic has been tempered by the challenges of recombinant TRAIL's short half-life (∼30 min), limited bioavailability and poor pharmacokinetics. The clinical trials of recombinant soluble TRAIL and agonistic TRAIL-R antibodies have thus far shown limited therapeutic benefit [9].

We and others have used mesenchymal stromal cells (MSCs) as a vector to target TRAIL therapy directly to tumor metastases [10–13]. MSCs preferentially migrate to and incorporate within tumors and their metastases-forming tumor stroma [10,14–18]. Several groups, including our own, have demonstrated that intravenously delivered MSCs preferentially localize in lung, breast and melanoma lung metastases [10,19,20], Kaposi's sarcoma [21], colorectal cancer [22] and glioma [23]. MSC tumor tropism has also been demonstrated after intraperitoneal delivery of MSCs for ovarian cancer [24] and intracerebral delivery of MSCs in a glioma model [25].

MSC-delivered targeted TRAIL overcomes the limited half-life of systemically delivered recombinant TRAIL. In murine models, we have shown that systemic injection of MSCs expressing full-length human TRAIL leads to a reduction in subcutaneous tumor growth and reduced, or indeed eliminated, lung metastases [10], and attenuates malignant pleural mesothelioma development [13]. Others have shown that MSCs engineered to express soluble TRAIL are able to kill cancer cells both *in vitro* and *in vivo* [26,27]. MSCs expressing soluble TRAIL may have an advantage *in vivo* in secreting TRAIL throughout the tumor rather than relying on the cell-cell contact that is required by the membrane-bound full-length TRAIL expressed on the MSC surface. In our preclinical development of MSC TRAIL therapy work, we wished to define the relative sensitivity of cancer cells to the different TRAIL forms expressed from a clinically approved lentiviral backbone. To elucidate which strategy is optimal, we created MSCs expressing full-length or soluble TRAIL and compared their activity in inducing cancer cell apoptosis.

## Methods

### Cell culture

Cell culture reagents were purchased from Invitrogen unless otherwise stated. Twenty cancer cell lines were used, including six lung cancer lines, A549, NCI-H460, NCI-H727, NCI-H23, H226 and PC9; seven malignant pleural mesothelioma lines, NCI-H2052, H2795, H2804, H2731, H2810, H2452 and H2869; three colon cancer lines, Colo205, HT29 and RKO; two renal cancer lines, RCC10 and HA7-RCC; one human oral squamous cell carcinoma line, H357; and one human breast adenocarcinoma line, MDAMB231 (M231). A549, H357 and M231 were obtained from Cancer Research United Kingdom. Other cell lines were kind gifts from Dr Ultan McDermott of the Wellcome Trust Sanger Institute, Cambridge, United Kingdom. NCI-H23, HT29 and Colo205 cells were cultured in Roswell Park Memorial Institute–1640 medium with 10% fetal bovine serum (FBS); RKO cells were cultured in Dulbecco's modified Eagle's medium (DMEM)/F-12 with 10% FBS; H357 cells were cultured in DMEM/F-12 (3:1) supplemented with 0.5 μg/mL hydrocortisone and 10^−10^ mol/L cholera toxin (Sigma-Aldrich), 10 ng/mL epithelial growth factor (Cambridge Biosciences) and 5 μg/mL human insulin (MP Biomedicals); all other cell lines were grown in the DMEM containing 10% FBS. Well-characterized human adult MSCs (passage 1) were purchased from the Texas A&M Health Science Center and cultured in the α-minimum essential medium containing 17% FBS.

### Construction of TRAIL vectors

The construction of the lentiviral vectors for the expression of flT and its soluble form (sT) was based on the lentiviral plasmid pCCL-c-Fes-Gfp [28]. The promoter of the backbone plasmid was replaced by the cytomegalovirus (CMV) promoter/enhancer [29] at XhoI and BamHI restriction sites. The CMV promoter/enhancer was amplified by means of polymerase chain reaction (PCR) with the use of the pCMV–dR8.74 plasmid as a template (a kind gift from Dr Thrasher, University College London). To create the flT vector, the flT-encoding complementary DNA (cDNA) was amplified by means of PCR with the use of our previously constructed inducible flT plasmid [10] as a template and inserted into the backbone in place of the green fluorescent protein (GFP) sequence through the use of BamHI and SalI sites; the resulting new plasmid is designated pCCL-CMV-flT. To create the sT vector, an open reading frame encoding an N-terminal–truncated extracellular portion of human TRAIL (amino acids 95–281) was amplified by means of PCR, which was then used as template for sequential PCRs to fuse the isoleucine zipper (IZ) (MKQIEDKIEEILSKIYHIENEIARIKKLIGERE) [30] in-frame and the murine immunoglobulin К-chain (IgК; 5′-ATGGAGACAGACACACTCCTGCTATGGGTACTGCTGCTCTGGGTTCCAGGTTCCACTGGTGAC-3′) leader sequence [31] to its N-terminal. The obtained sT sequence was inserted into the pCCL-CMV-flT in place of flT through the BamHI and SalI sites, creating the sT vector designated pCCL-CMV-sT.

### Lentivirus preparation and transduction of MSCs

The lentivirus supernatants were produced by co-transfection of 293T cells with construct plasmids together with the packaging plasmids pCMV-dR8.74 and pMD2.G in the presence of a DNA transfection reagent jetPEI (Source Bioscience UK Ltd). The pMD2.G and pCMV-dR8.74 plasmids were kindly provided by Dr Thrasher, University College London (UCL). Lentiviruses in supernatants were concentrated by ultracentrifugation at 17,000 rpm (SW28 rotor, Optima LE80K Ultracentrifuge, Beckman) for 2 h at 4°C. Human MSCs were transduced with a multiplicity of infection (MOI) of 2 and 8 μg/mL polybrene (Sigma-Aldrich). Human TRAIL expression was verified by means of an enzyme-linked immunoassay (ELISA) (R&D Systems) according to the manufacturer's instructions and by Western blot analysis.

### Flow cytometry of lentivirus-transduced cells

To determine the titers of prepared lentiviruses, 293T cells were transduced with serial dilutions of viruses in the presence of 8 μg/mL polybrene. After 3 days, TRAIL or GFP expression was examined by means of flow cytometry. For flow cytometry detection of TRAIL expression, cells were stained with a 1:10 dilution of phycoerythrin (PE)-conjugated mouse monoclonal antibody against human TRAIL (Ab47230, Abcam).

### MSC phenotyping and differentiation assay

MSC phenotyping was carried out by use of the human MSC Phenotyping Kit (Miltenyi Biotec, Cat. No. 130–095–198) according to the manufacturer's instructions, and cells were analyzed by means of flow cytometry. Differentiation of passage 7 MSCs with or without transduction was performed by use of the StemPro Chondrogenesis, Osteogenesis or Adipogenesis Differentiation Kits (GIBCO Invitrogen Cell Culture). Adipocytes were stained with high content screening LipidTOX green and 4′-6-diamidino-2-phenylindole (DAPI), osteocytes were stained with alizarin red S and the chondrogenic pellet was stained with alcian blue, all according to the manufacturer's instructions. In addition, to quantitatively compare the differentiation potential of transduced MSCs with untransduced parental cells, MSCs and MSC-flT cells with or without induction of adipogenesis differentiation for 10 days or with/without induction of osteogenesis for 13 days were analyzed for differentiation marker gene expression by means of real-time quantitative PCR. In brief, total cellular RNAs were extracted and reverse-transcribed into cDNAs, followed by real-time PCR analysis with the use of SYBR green PCR Master Mix (AB Inc, P/N 4367659) according to the manufacturer's instructions. The previously validated MSC adipogenesis marker gene PPARG [32] and osteogenesis gene BMP2 [33] were examined for their messenger RNA expression changes by use of the commonly used reference gene RPL13A for normalization, which has been validated as suitable for real-time quantitative PCR of bone-marrow–derived MSCs [34]. Primers were purchased for PPARG (Accession No. NM-015869/Cat. No. HP226175, OriGene Technologies), BMP2 (NM_001200/HP205130) and RPL13A (NM_012423/HP210356). Data analysis was performed by use of the previously described 2^−ΔΔCt^ method [35].

### Cell proliferation assays

Assessment of cell proliferation and viability was determined with the use of the XTT Cell Proliferation Assay Kit, according to the manufacturer's instructions; 10,000 passage 7 MSCs were seeded per well of a 24-well plate, and the assay was performed in triplicate every 24 h for a total of 7 days.

### Western blot analysis

Passage 4 or 5 MSCs were harvested and lysed in radio-immunoprecipitation buffer (phosphate-buffered saline, 1% Igepal Ca-630, 0.5% sodium deoxycholate and 0.1% sodium dodecyl sulfate [Sigma]) supplemented with complete protease inhibitor cocktail (Complete-mini; Roche Diagnostics); 10 μg of protein from the whole-cell lysates and 5 μL of 50-fold–concentrated culture supernatant were prepared, resolved on 4% to 12% polyacrylamide sodium dodecyl sulfate gels and analyzed by means of immunoblotting with rabbit anti-human TRAIL (c-terminal) Ab (ab42121, Abcam) and anti–α-tubulin Ab (11H10, Cell Signalling), respectively.

### Immunofluorescence

Localization of TRAIL in cells was examined by means of immunofluorescence staining. For intracellular staining, cells were grown on chamber slides for 2 days, fixed with 4% paraformaldehyde, permeabilized in 0.1% saponin-containing buffer, blocked in phosphate-buffered saline containing 10% FBS and 0.1% saponin and then stained with the PE-conjugated mouse anti-human TRAIL monoclonal antibody B-S23 (Abcam, Cat. No. ab47230). The counterstain Alexa-488–conjugated phalloidin (Life Technologies) was also added for labeling filamentous actin (F-actin). For cell-surface TRAIL labeling, cells were stained with PE-conjugated anti-TRAIL Ab after fixation and blocking but before permeabilization and F-actin counterstaining. Stained cells were mounted with the ProLong Gold Antifade Reagent with DAPI (Invitrogen), viewed and imaged by confocal microscopy (Leica TCS SP2 microscope).

### Supernatant TRAIL preparation

Two million transduced MSCs were cultured in one T175 flask for 3 days with 20 mL of media. Proteins were concentrated 50-fold through the use of the centrifugal concentration column (Millipore UFC901008, MWCO 10 kDa). TRAIL levels in concentrated media were determined with the use of the TRAIL quantification ELISA kit (R&D Systems).

### Co-culture and apoptosis analysis

DiI-labeled cancer cells (*n* = 8000) were plated into one well of a 96-well plate, to which transduced MSCs, purified recombinant TRAIL 50 ng/mL (amino acids 114–281) (Peprotech), the pan-caspase inhibitor Z-VAD-FMK (1 μg/mL [Sigma]), a neutralizing monoclonal anti-TRAIL Ab (10 ng/mL [Sigma, Cat. No. T3067]) or control medium was added for 24 h. Ratios of MSCs to cancer cells included 1:10, 2:10, 4:10, 6:10, 8:10 and 10:10 (MSC: cancer cell). Floating and adherent cells were stained with AF647-conjugated Annexin V (Invitrogen) and 2 μg/mL DAPI (Sigma) and were assessed by means of flow cytometry. Annexin V+ cells were considered to have undergone apoptosis; Annexin V+/DAPI+ cells were considered to be dead by apoptosis.

### Active caspase-8 staining

Cancer cells were DiO-labeled according to the manufacturer's instructions, before being treated for 24 h with MSCs expressing TRAIL or GFP to induce apoptosis. The treated cells were harvested, stained with the active caspase-8 inhibitor IETD-FMK-conjugated to sulfo-rhodamine (BioVision, K198-25) according to the manufacturer's instructions and analyzed by means of flow cytometry.

### Statistical analysis

Data were analyzed with the use of GraphPad Prism 6 software (GraphPad Software) and Mastercycler ep realplex software version 2.2. Statistical significance between groups was determined by use of the Student's *t*-test. Significant probability values are denoted as *P* < 0.05, *P* < 0.01.

## Results

### Construction of lentiviral vectors and TRAIL expression

**Figure 1:**
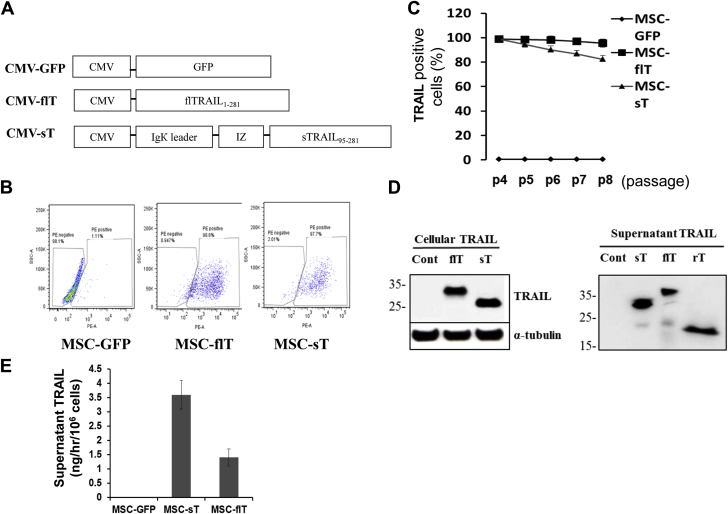
Expression of recombinant TRAIL by transduced MSCs. (A) Schematic description of TRAIL expression constructs. (B) Fluorescence-activated cell sorting analysis of lentivirus-transduced MSCs. (C) Long-term fluorescence-activated cell sorting analysis of TRAIL expression in MSCs transduced at passage 3 (p3), expanded and passaged every 7 days until p8. (D) Detection of TRAIL and α-tubulin expression by immunoblotting. Cont. represents MSC-GFP cell lysates or supernatant medium; flT and sT represent cell lysates or concentrated culture supernatant from MSC-flT and MSC-sT cells, respectively; rT represents 1 ng of purified recombinant human TRAIL (amino acids 114–281) produced from bacterial cells (PeproTech). (E) Levels of TRAIL in cell culture supernatants from MSCs transduced at p3 and expanded for one passage, measured by ELISA. Data presented as TRAIL released by 1 million cells per hour (ng/h/1 × 10^6^ cells). Data represent averages ± SEM (*n* = 5).

The lentiviral plasmid pCCL-c-Fes-Gfp [28] was used to construct two lentiviral vectors, pCCL-CMV-flT (full-length human TRAIL) and pCCL-CMV-sT (truncated soluble TRAIL). The c-Fes promoter in the backbone vector was replaced by a CMV promoter/enhancer to give constitutive and high expression of proteins of interest. For the flT construct, the GFP sequence was replaced with human TRAIL (amino acids 1–281). The soluble TRAIL vector was made by fusing in-frame DNA sequences (from 5′ to 3′), including a leader sequence from murine immunoglobulin К-chain (IgК leader) to assist secretion, an IZ to enhance trimerization and amino acids 95–281 of human TRAIL (Figure 1A). TRAIL lentiviruses were prepared by transfecting 293T cells with TRAIL constructs together with packing plasmids.

Well-characterized human adult MSCs were purchased from the Texas A&M Health Science Center and were shown to be able to differentiate into chondrogenic, osteogenic and adipogenic lineages. TRAIL-transduced MSCs (MOI 2) were examined by flow cytometry with the use of a PE-conjugated anti-human TRAIL antibody, which demonstrated that more than 98% of flT-transduced MSCs (MSC-flT) were positive for TRAIL expression, whereas only approximately 1% of control GFP virus-infected cells were positive, which indicated TRAIL expression was not the result of endogenous TRAIL induction after lentivirus infection (Figure 1B). In addition, more than 97% of soluble TRAIL-transduced MSCs (MSC-sT) were positive for TRAIL expression (Figure 1B). Of note, full-length TRAIL expression was stable through passages 4–8, but expression of soluble TRAIL decreased by just under 20% during this time (Figure 1C). All further MSC TRAIL comparison experiments were carried out in passage 4 or 5 cells.

To further assess MSC TRAIL expression, immunoblot analysis of cellular lysates was carried out. Both MSC-flT and MSC-sT cells expressed abundant cellular TRAIL proteins with similar expression levels, whereas GFP-transduced MSCs showed no detectable TRAIL expression (Figure 1D). The MSC-flT lysate showed a TRAIL band of ∼32 kDa and the MSC-sT lysate showed a band of ∼27 kDa, which was larger than its predicted size of 24 kDa, possibly as a result of the glycosylation of the ligand [1]. Secreted TRAIL protein was detected in supernatants of both MSC-sT and MSC-flT cells but not in that of GFP-transduced cells (Figure 1E). The MSC-sT cells secreted abundant soluble TRAIL of ∼27 kDa and ∼24 kDa size. Three soluble TRAIL molecular forms were detected in the supernatant of MSC-flT cells, that of ∼35 kDa and ∼32 kDa, corresponding to the glycosylated and non-glycosylated full-length TRAIL, and that of ∼24 kDa, corresponding to a cleaved form (Figure 1D) [36].

To assess the rate of TRAIL secretion, a highly specific sandwich ELISA was used to quantify soluble TRAIL in MSC supernatant medium. MSC-sT cells secrete high levels of soluble TRAIL at a rate of 3.63 ± 0.71 ng/h for every 1 million cells (Figure 1E). Interestingly, MSC-flT cells secrete 1.3 ± 0.52 ng/h soluble TRAIL for every 1 million cells. MSC-GFP cells did not secrete measurable TRAIL (Figure 1D, E).

**Figure 2:**
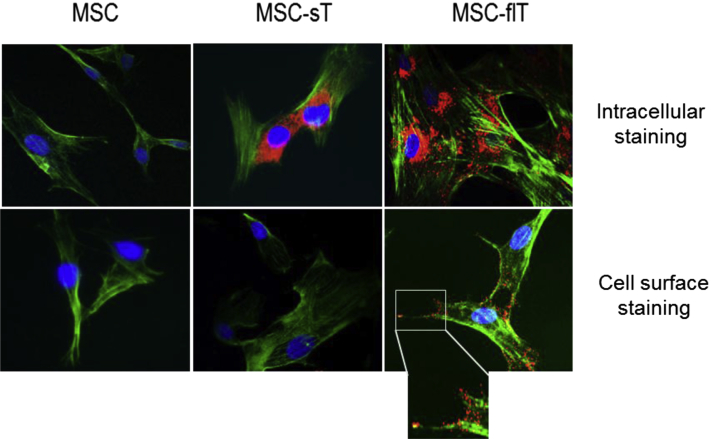
Immunofluorescent detection of recombinant TRAIL (red) expressed by transduced MSCs. Phalloidin staining was used to show filamentous actin (green); nuclei were labeled with DAPI (blue). Top panel shows intracellular staining; bottom panel shows cell surface staining. Images are representative of at least three experiments for each staining condition. Magnification ×400.

To examine the cellular distribution of TRAIL in transduced MSCs, immunolabeling of the ligand was performed with the use of a PE-conjugated anti-TRAIL antibody. As shown in Figure 2, positive staining of TRAIL was observed in both MSC-flT and MSC-sT cells but not in parental untransduced cells. Fluorescence microscopy revealed that TRAIL distribution is exclusively cytoplasmic in MSC-sT cells, whereas MSC-flT cells show both cell-surface and intracellular TRAIL expression. Interestingly, TRAIL labeling in MSC-flT cells appeared to be enriched at the leading edge of lamellipodia and tips of filopodia (Figure 2, inset).

### MSC viability, protein expression and differentiation are not affected by TRAIL-expressing lentiviral infection

**Figure 3:**
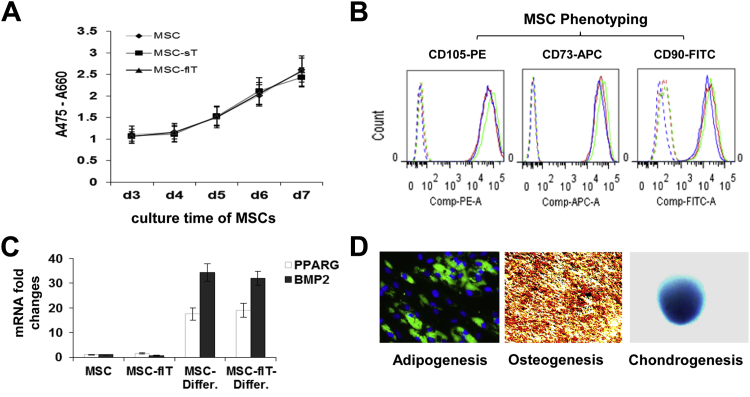
TRAIL expression by lentiviral transduction does not affect MSC viability, proliferation, marker protein expression or differentiation potential. (A) Cell viability and proliferation were assessed with the use of the XTT assay for 7 days after transduction. (B) Phenotyping of cultured MSCs for expression of conventional MSC markers is shown. Dashed line shows isotype antibody control; solid line shows marker-specific antibody staining (red, MSC; blue, MSC-flT; green, MSC-sT). (C) MSC differentiation potential was assessed by means of real-time quantitative PCR for adipogenic marker gene PPARG and osteogenic gene BMP2 before and after differentiation period (Differ.) and immunochemistry. (D) High content screening lipidTOX green staining (green) for neutral lipid and DAPI staining for nuclei (blue) to show adipogenic differentiation; middle, alizarin red S staining (red) to show osteogenic differentiation; right, alcian blue staining (blue) to show chondrogenic differentiation. Magnification ×200 for adipogenesis and osteogenesis assays; magnification ×50 for chondrogenesis assay.

TRAIL-transduced MSCs were analyzed for changes in phenotype after lentiviral infection. MSC-sT and MSC-flT cells demonstrated viability and proliferation rates that were equivalent to non-transduced cells (Figure 3A). Expression of the characteristic MSC markers CD105, CD90 and CD73 (Figure 3B) and lack of expression of the haematopoietic markers CD14, CD20, CD34 and CD45 (not shown) was unchanged by transduction. Differentiation potential of lentivirally infected cells was also unaltered compared with parent untransduced cells, with both cell types showing similar transcriptional expression increases of the adipogenesis marker gene PPARG and the osteogenesis marker gene BMP2 (Figure 3C) as well as equivalent adipogenic, osteogenic and chondrogenic differentiation capacities (MSC-flT shown in Figure 3D) after differentiation induction.

### MSC-flT cells induce greater apoptosis in cancer cells than MSC-sT cells

In Figure 1E, we demonstrate that both MSC-sT and MSC-flT cells secrete TRAIL into their culture medium. However, the level of TRAIL that is secreted by MSC-sT cells is higher than that secreted by MSC-flT cells, which indicates that these cells may have higher cancer cell-killing efficiency. To test this, we examined cancer cell apoptosis in co-culture experiments with MSC-sT and MSC-flT cells. We initially examined killing of the known TRAIL-sensitive MDAMB231 (M231) cell line and then tested the TRAIL-resistant lung cancer line, A549.

**Figure 4:**
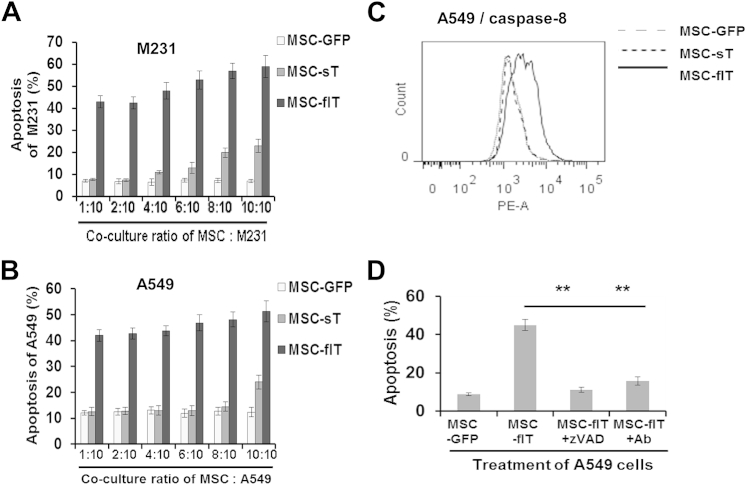
MSCs expressing TRAIL induce apoptosis in cancer cells. (A, B) Cancer cell apoptosis was measured by means of flow cytometry 24 h after co-culturing the breast adenocarcinoma cells MDAMB231 (M231) (A) or the lung adenocarcinoma cells A549 (B) with MSC-GFP, MSC-flT or MSC-sT cells, with an increasing ratio of MSCs to cancer cells in the co-culture system. (C) Activated caspase-8 levels in A549 cells were measured by means of flow cytometry after co-culture with MSC-GFP, MSC-sT or MSC-flT cells at a ratio of 4:10 (MSC: cancer cell). (D) MSC-flT–induced cancer cell apoptosis can be blocked by the 20 μmol/L pan-caspase inhibitor Z-VAD-FMK (zVAD) and 100 ng/mL TRAIL-neutralizing monoclonal Ab (T3067, Sigma-Aldrich). Data represent averages ± SEM. ∗∗*P* < 0.01 compared with MSC-flT co-culture by Student's *t*-test.

M231 cells were co-cultured with MSC-sT, MSC-flT or MSC-GFP cells at ratios of increasing numbers of MSCs to cancer cells, ranging from 1:10 to 1:1 (MSC: cancer cell). Apoptosis was measured by use of DAPI and Annexin V labeling by means of flow cytometry. As shown in Figure 4A, both MSC-sT and MSC-flT cells induced M231 apoptosis in a dose-dependent manner; however, MSC-flT cells were more efficient than were MSC-sT cells at inducing apoptosis (Figure 4A). At all co-culture cell number ratios, MSC-flT cells showed higher killing capacity than did MSC-sT cells. Similarly, A549 cells demonstrated greater cell death when co-cultured with MSC-flT cells compared with MSC-sT cells (Figure 4B). TRAIL-induced apoptosis involves caspase-8 recruitment and activation [37]. Thus, we next analyzed the activated caspase-8 levels in co-cultured cancer cells by means of flow cytometry. The A549 cells showed increased caspase-8 activation when co-cultured with MSC-flT cells at a ratio of 4:10 (MSC: cancer cell) but not when cultured with MSC-sT cells or with MSC-GFP cells (Figure 4C). This is consistent with the relative cancer cell apoptosis rates (Figure 4B). To further confirm apoptotic pathway activation by TRAIL-expressing MSCs, the caspase inhibitor Z-VAD-FMK and the anti-TRAIL antibody T3067 were added to MSC-flT cell co-cultures. These experiments demonstrated that MSC-flT killing is caspase-dependent and requires TRAIL receptor activation (Figure 4D).

### MSC-flT cells partly overcome TRAIL resistance of cancer cells

To determine if the higher cancer cell-killing capacity exerted by MSC-flT cells compared with MSC-sT cells is more broadly applicable, we extended our co-culture assay to a panel of 20 established cancer cell lines. The panel included six cancer types consisting of six lung cancer lines, A549, NCI-H460, NCI-H727, NCI-H23, H-226 and PC9; seven malignant mesothelioma lines, NCI-H2052, H2795, H2804, H2731, H2810, H2452 and H2869; three colon cancer lines, Colo205, HT29 and RKO; two renal cancer lines, RCC10 and HA7-RCC; one human oral squamous cell carcinoma line, H357; and one breast cancer line, MDAMB231.

**Figure 5:**
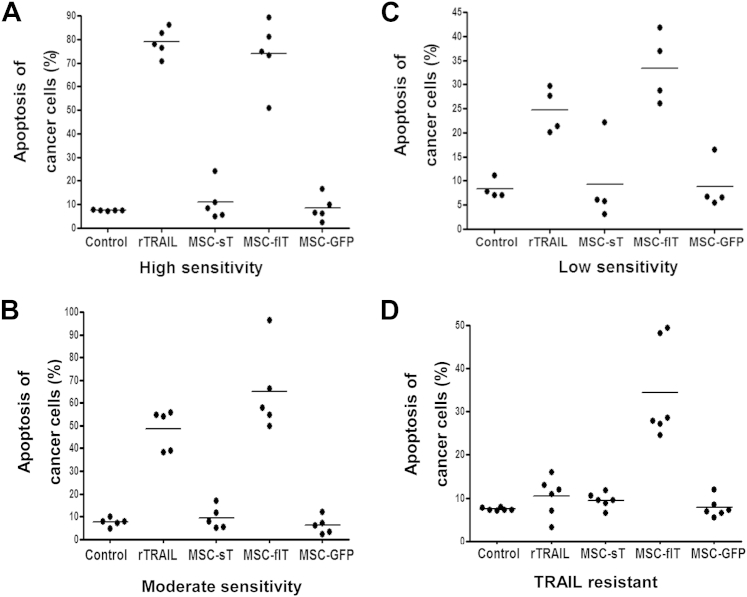
MSC-flT cells induce cancer cell apoptosis with a higher efficiency than MSC-sT cells. (A–D) Apoptosis of five highly TRAIL-sensitive cancer cell lines, Colo205, NCI-H460, H727, H2795 and H2804 (A); five moderately TRAIL-sensitive cancer cell lines, H2731, H226, H2869, PC9 and M231 (B); four cancer cell lines of low TRAIL sensitivity, HT29, H357, H2452 and RKO (C); and six TRAIL-resistant cancer cell lines, A549, NCI-H2052, H2810, NCI-H23, RCC10 and HA7-RCC (D) was determined after 24 h of culture with medium (control), 50 ng/mL of purified recombinant TRAIL (rTRAIL), MSC-GFP, MSC-flT or MSC-sT, with a ratio of 4 p4 MSC to 10 cancer cells. Data represent averages of three experiments with triplicate repeats for each cell line.

The 20 cancer cell lines were also treated with recombinant TRAIL (rTRAIL) at a concentration of 50 ng/mL or with control GFP-transduced MSCs. Control MSCs did not induce cancer cell apoptosis. After rTRAIL treatment, the 20 cell lines showed a varied response and were grouped accordingly into those that were rTRAIL-sensitive (apoptosis ≥70%; five cell lines), those that were moderately TRAIL-sensitive (apoptosis 35% to 70%; five cell lines), those that showed low TRAIL sensitivity (apoptosis 20% to 35%; four cell lines) or those that were TRAIL-resistant (apoptosis ≤20%; six cell lines) (Figure 5A–D). Control MSC-GFP, MSC-flT and MSC-sT cells were applied to cancer cells with a ratio of 4:10 (MSC: cancer cell). For all four cancer cell groups, MSC-sT cells showed only marginal effects on apoptosis induction, whereas MSC-flT cells demonstrated effective killing of cancer cells (Figure 5A–D). Of note, in the highly TRAIL-sensitive group, MSC-flT cells exerted a similar level of cancer cell killing to 50 ng/mL rTRAIL; for moderate and low TRAIL-sensitive and TRAIL-resistant groups, MSC-flT cells induced more apoptosis than did rTRAIL.

### Soluble TRAIL released by MSC-flT cells is of higher activity than rTRAIL and sT

Unexpectedly, MSC-sT and MSC-flT cells both release abundant TRAIL into the supernatant medium (Figure 1E). We had anticipated that the truncated soluble form of TRAIL that is secreted by MSC-sT cells would have pro-apoptotic effects on nearby cancer cells. Having found that MSC-flT cells not only express full-length TRAIL on their cell surface but also secrete full-length TRAIL into the culture medium, we tested the relative killing efficacy of both the full-length and the truncated secreted forms of TRAIL.

To obtain sufficient amounts of soluble TRAIL, supernatant media were collected from cultured MSC-flT and MSC-sT cells under low serum conditions (1% FBS), filtered through 0.2-μm filters and concentrated 50-fold with centrifugal columns (10 kDa). Supernatant medium from GFP-transduced MSCs was also collected and concentrated and used as a control. ELISA quantification showed that the concentrated MSC-flT supernatant medium contained 0.4 ± 0.2 μg/mL of TRAIL and that the MSC-sT supernatant medium contained 1.2 ± 0.3 μg/mL TRAIL.

**Figure 6:**
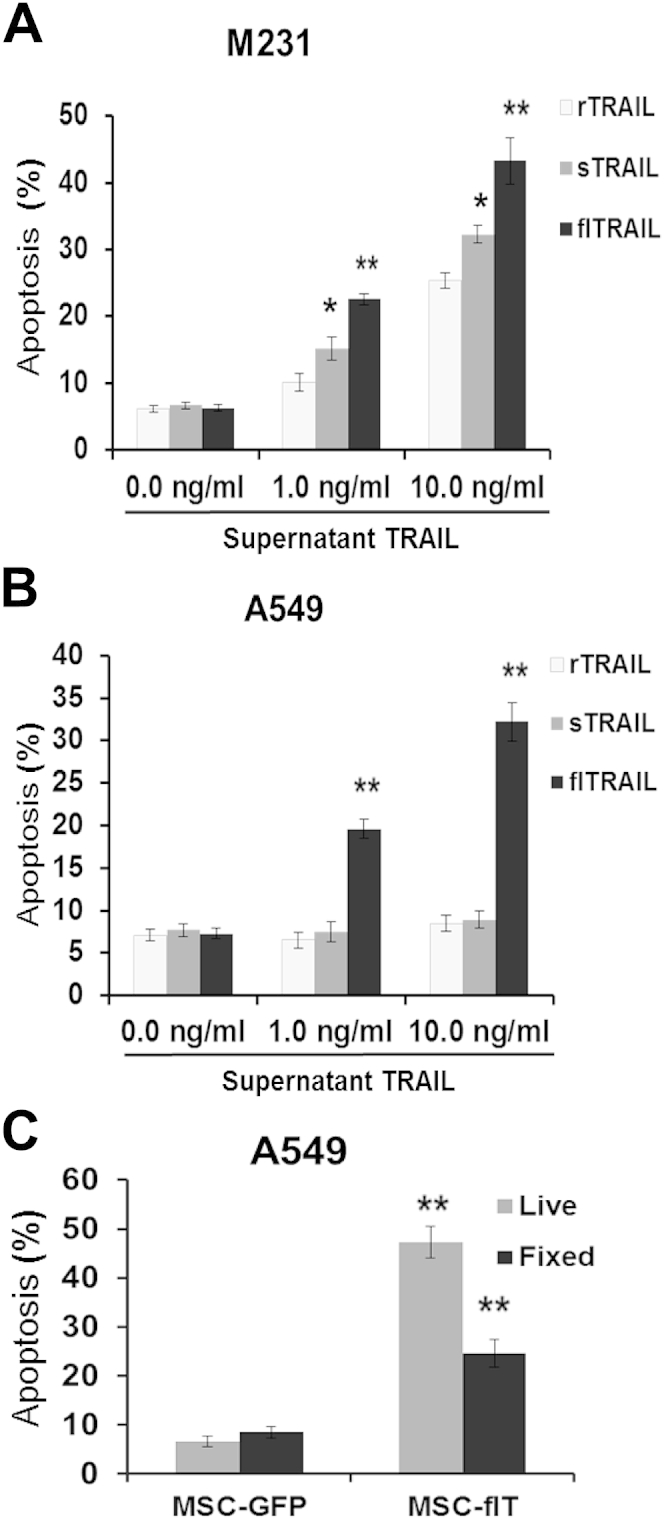
MSC-flT–derived cell surface and secreted TRAILs contribute to induction of apoptosis in cancer cells. (A, B) Apoptosis was measured by means of flow cytometry after exposure of M231 cells (A) or A549 cells (B) for 24 h to increasing doses of recombinant TRAIL (rTRAIL), supernatant TRAIL from MSC-sT (sTRAIL) or supernatant TRAIL from MSC-flT (flTRAIL). (C) Apoptosis was measured after 24-h co-culture of A549 cells with live or fixed MSC-GFP and MSC-flT cells, respectively. Data represent averages ± SEM (*n* = 3). ∗*P* < 0.05, ∗∗*P* < 0.01 compared with rTRAIL and MSC-GFP treatment, respectively, by Student's *t*-test.

Primary human lung bronchial epithelial cells and two cancer cell lines, M231 and A549, were treated with the supernatant preparations of flT or sT, rTRAIL or control medium to compare cytotoxicity. Human lung bronchial epithelial cells showed complete resistance to all three TRAIL preparations (data not shown). By contrast, M231 cells, which are a moderate TRAIL-sensitive cancer cell line, showed dose-dependent sensitivity to all three TRAIL forms (Figure 6A). As expected, as a result of our insertion of the trimerization domain IZ, sT showed higher cytotoxicity than did rTRAIL; however, interestingly, flT induced most apoptosis (Figure 6A). Of particular note, flT was capable of inducing apoptosis in rTRAIL-resistant A549 cells, but sT was not capable (Figure 6B).

### Cell-surface TRAIL on MSC-flT cells contributes to apoptosis induction

To confirm the role of cell-surface TRAIL expression in the induction of apoptosis, we fixed MSC-flT cells with 4% paraformaldehyde to stop TRAIL secretion into the supernatant and then co-cultured them with A549 cells. Whereas fixed-control MSC-GFP cells did not show any cancer cell killing, the fixed MSC-flT cells demonstrated significant killing of cancer cells (Figure 6C). TRAIL ELISA confirmed no detectable TRAIL release in the supernatant of fixed MSC-flT cells. Therefore, the induction of apoptosis by MSC-flT cells is at least partially due to the TRAIL expressed on the cell surface. MSC-sT cells were also fixed and tested for A549 killing but failed to show any cancer cell killing (data not shown), which is consistent with the fact that MSC-sT cells do not express cell-surface TRAIL (Figure 2).

## Discussion

We have previously shown that MSCs expressing full-length TRAIL can significantly reduce, and in some cases eliminate, lung metastases in a murine model [10]. In these previous experiments, we used the pRRL-derived self-inactivating lentiviral backbone [38], into which the TRAIL-encoding cDNA and IRES-eGFP reporter sequence were sequentially introduced, under the control of the tetracycline-regulated CMV minimal promoter [10]. To produce a clinically relevant therapeutic, in this study we subcloned soluble or full-length TRAIL and CMV promoter/enhancer into the MHRA human–approved lentiviral vector pCCL-c-Fes-Gfp (30). The new vectors that we created use the constitutive CMV promoter and do not contain the tet-on conditional system. We then compared the killing efficiency of full-length TRAIL with soluble TRAIL, with the hypothesis that secreted TRAIL may improve efficiency.

In the present study, we have shown that MSC infection with the new lentiviruses expressing the different TRAIL forms does not affect MSC differentiation capacity or cell proliferation. We demonstrate that full-length TRAIL is indeed expressed at the cell membrane, in contrast to the cytoplasmic expression of soluble TRAIL. Intriguingly, both soluble and full-length TRAIL are secreted (although soluble TRAIL is secreted at greater levels), which removes some of the potential benefit of the use of a soluble TRAIL to treat an *in vivo* tumor in which TRAIL diffusion through the tumor could enhance anti-cancer activity. Most importantly, we show that cellular presentation of the full-length form of TRAIL is superior in cancer cell killing to the cellular production of the soluble form of TRAIL.

MSCs have been successfully used to deliver several gene therapies to tumors in murine models. Cytokines and chemokines such as interleukin-2 (IL-2) [39], IL-12 [40], CX3CL1 [20] and interferon-β [19,41–43] have been shown to be therapeutically beneficial when delivered by MSCs. In addition, MSCs were successfully used to deliver oncolytic virus [22,44]. MSCs have also been engineered to express an enzyme that converts a pro-drug into a cytotoxic agent at the site of tumors [45,46]. MSCs engineered to express TRAIL have shown therapeutic effects in mouse lung metastasis [10,11] and glioma [12] models. We recently demonstrated that intravenously delivered MSCs expressing TRAIL can home into malignant pleural mesothelioma and can induce apoptosis in cancer cells [13].

MSCs do not normally express endogenous TRAIL (Figure 1) [47] and, in the present study, we show that the infection with a lentivirus expressing GFP does not lead to any detectable endogenous TRAIL expression (Figure 1). Interestingly, TNF-α was found to be able to trigger endogenous TRAIL expression in MSCs [47]. However, TNF-α–induced TRAIL expression appeared to only occur within cells and cells lacked apparent soluble TRAIL release, which necessitates cell-to-cell contact for apoptotic activity [47].

TRAIL and other members of the TNF superfamily are type II transmembrane proteins and are expressed as membrane-bound homotrimeric molecules. It has been demonstrated by others that oligomerization is necessary for efficient induction of apoptosis in target cells. Holler *et al.* [48] showed that a hexameric FasL, consisting of two homotrimers at close proximity, represents the minimal ligand complex structure that is required to effectively form the death-inducing signaling complex and to activate apoptosis. The trimeric FasL failed to induce a death-inducing signaling complex and is thus inefficient in triggering apoptosis. A recent study found that TRAIL proteins expressed by human syncytiotrophoblasts exist as a hexameric form on exosome membranes [49]. The particulate aggregation of flT that we have observed on the cell surface of MSCs and the higher biological activity of flT than IZ-fused soluble TRAIL both indicate the possibility that higher-order oligermization of flT occurs on the cell membrane of transduced MSCs.

In our construction of soluble TRAIL, we used an IZ as a trimerization enhancer, which has been previously shown to facilitate the formation of a TRAIL homotrimer and to enhance its cytotoxicity [50]. The IZ-fused sT construct created in this study confirmed the secretory advantage of the use of IZ for recombinant soluble TRAIL preparation. However, we demonstrate that flT is also secreted and is more efficient in inducing apoptosis in cancer cells than either rTRAIL produced from bacterial cells or the trimerized sT secreted by transduced MSCs. To our knowledge, this is the first observation suggesting that mammalian cell-secreted flT is superior to N-terminal-truncated soluble TRAIL versions (amino acids 114–281 or amino acids 95–281).

An important observation is that MSC expression of flT induces apoptosis in cancers that are completely resistant to recombinant soluble TRAIL. The reason for this effect is not clear; however, two recent publications outline that higher-order clustering of TRAIL-Rs may be necessary for full extrinsic death pathway activation [51,52]. These studies demonstrate that neither soluble TRAIL nor antibodies against TRAIL-Rs are capable of higher-order clustering alone, although they are in combination. TRAIL expressed on the cell surface may be able to induce higher-order clustering as a result of movements permitted by the fluidic nature of the cell membrane. This might be the reason for the greater efficacy of MSC-flT than recombinant TRAIL or MSC-sT in our *in vitro* studies. It will be interesting to understand in future studies whether flT but not sT is capable of this clustering and whether TRAIL requires cell membrane binding for this full killing effect.

Soluble recombinant TRAIL has been extensively tested as a cancer therapy *in vitro* and in human studies [4,5,53–60]. Completed clinical trials have used recombinant protein doses of up to 30 mg/kg [59], possibly because of its short half-life and the low efficiency of the recombinant soluble ligand. However, therapeutic benefits have been limited [9]. The clinical failure of recombinant soluble TRAIL has mainly been attributed to cancer cell resistance [9]. MSC delivery of full-length TRAIL not only results in the delivery of stably expressed TRAIL but also overcomes at least some of the resistance.

In the present study, we have therefore validated cell infection, target gene expression and gene function through the use of our clinically approved lentiviral backbone with the constitutive CMV promoter. We show that transduction with this virus does not adversely affect cell phenotype and we clearly demonstrate that the cancer cell-killing function of full-length TRAIL is superior to that of the shortened soluble form of TRAIL. In further studies, it will be interesting to delineate whether this improved function of full-length TRAIL is due to higher order clustering. Importantly, we also show that MSC-flT cells are capable of partially overcoming cancer cell TRAIL resistance and that they hold promise for the treatment of diverse cancer types.
